# Kyungheechunggan-Tang-01, a New Herbal Medication, Suppresses LPS-Induced Inflammatory Responses through JAK/STAT Signaling Pathway in RAW 264.7 Macrophages

**DOI:** 10.1155/2017/7383104

**Published:** 2017-11-29

**Authors:** Hee-Soo Han, Eungyeong Jang, Ji-Sun Shin, Kyung-Soo Inn, Jang-Hoon Lee, Geonha Park, Young Pyo Jang, Kyung-Tae Lee

**Affiliations:** ^1^Department of Pharmaceutical Biochemistry, College of Pharmacy, Kyung Hee University, Kyungheedae-ro, Dongdaemun-gu, Seoul 02447, Republic of Korea; ^2^Department of Life and Nanopharmaceutical Sciences, College of Pharmacy, Kyung Hee University, Kyungheedae-ro, Dongdaemun-gu, Seoul 02447, Republic of Korea; ^3^Department of Internal Medicine, College of Korean Medicine, 26 Kyungheedae-ro, Dongdaemun-gu, Seoul 02447, Republic of Korea; ^4^Department of Pharmaceutical Science, College of Pharmacy, Kyung Hee University, Kyungheedae-ro, Dongdaemun-gu, Seoul 02447, Republic of Korea; ^5^Department of Oriental Pharmaceutical Sciences, College of Pharmacy, Kyung Hee University, 26 Kyungheedae-ro, Dongdaemun-gu, Seoul 02447, Republic of Korea

## Abstract

Medicinal plants have been used as alternative therapeutic tools to alleviate inflammatory diseases. The objective of this study was to evaluate anti-inflammatory properties of Kyungheechunggan-tang- (KCT-) 01, KCT-02, and Injinchunggan-tang (IJCGT) as newly developed decoctions containing 3–11 herbs in LPS-induced macrophages. KCT-01 showed the most potent inhibitory effects on LPS-induced NO, PGE_2_, TNF-*α*, and IL-6 production among those three herbal formulas. In addition, KCT-01 significantly inhibited LPS-induced iNOS and COX-2 at protein levels and expression of iNOS, COX-2, TNF-*α*, and IL-6 at mRNA levels. Molecular data revealed that KCT-01 attenuated the activation of JAK/STAT signaling cascade without affecting NF-*κ*B or AP-1 activation. In ear inflammation induced by croton oil, KCT-01 significantly reduced edema, MPO activity, expression levels of iNOS and COX-2, and STAT3 phosphorylation in ear tissues. Taken together, our findings suggest that KCT-01 can downregulate the expression of proinflammatory genes by inhibiting JAK/STAT signaling pathway under inflammatory conditions. This study provides useful data for further exploration and application of KCT-01 as a potential anti-inflammatory medicine.

## 1. Introduction

Inflammation is a complicated biological response caused by many harmful stimuli, including pathogens, bacteria, and irritants [1]. It is also a tissue protective reaction of immune cells including macrophages. Initiation of inflammation is triggered by interactions between surface receptors such as Toll-like receptor- (TLR-) 4 and TLR-2 and their ligands, including lipopolysaccharides (LPS) derived from bacteria [2]. When activated by LPS, macrophage as a potent immune system activator can induce large amounts of proinflammatory mediators, nitric oxide (NO), prostaglandin E_2_ (PGE_2_), and cytokines such as interleukin- (IL-) 1*β*, IL-6, and tumor necrosis factor-*α* (TNF-*α*). These proinflammatory mediators and cytokines can lead to inflammation and various clinical manifestations [3]. Overexpressed proinflammatory mediators further exacerbate immune responses in many acute and chronic inflammatory diseases, including arteriosclerosis, inflammatory bowel disease, arthritis, infectious diseases, and cancer [4]. Accordingly, materials or compounds that can inhibit these proinflammatory mediators have been considered as potential anti-inflammatory agents.

Janus kinase- (JAK-) signal transducer and activator of transcription (STAT) cascade is a critical inflammatory signaling pathway that mediates immune responses [5]. In particular, STATs have been reported to play a pivotal role in inflammatory signaling cascades triggered by LPS and several cytokines such as interferon gamma (IFN-*γ*) [6–8]. TLR-4 activation results in phosphorylation of receptor-associated enzymes JAKs known to be activators of STATs [9]. Binding of ligands to their receptors leads to phosphorylation of JAKs, which will induce phosphorylation of STATs that in turn leads to the release of STATs from the receptor complex. Released STATs can form homo- or heterodimers and translocate into the nucleus to regulate transcription of target genes encoding proinflammatory cytokines and inducible enzymes such as inducible nitric oxide synthase [1] and cyclooxygenase-2 (COX-2) [10–12]. Since JAK-STAT signaling pathway is involved in the mediation of proinflammatory gene expression, it is important to tightly regulate its activity to prevent inflammatory responses.

Viral infection, high consumption of alcohol, fat accumulation, and toxic agent are the main causes of chronic inflammation in the liver [13]. Chronic inflammation causes pathological changes in liver function and, therefore, might progress severe problems such as liver fibrosis and cancer [14]. Thus, controlling inflammatory responses in the liver is desirable in the management of severe hepatic diseases. Many traditional herbal medicines have been revealed to have pharmacological properties against inflammatory injury [15]. Injinchunggan-tang (IJCGT) is a herbal medicine containing 11 medicinal herbs. It has been widely used to treat hepatic diseases in Kyung Hee University Korean Medicine Hospital (Seoul, Republic of Korea) [16–21]. Based on IJCGT, we reorganized new herbal medications called Kyungheechunggan-tang- (KCT-) 01 which contained* Artemisia Capillaris Herba, Sanguisorbae Radix, *and* Curcuma longa Radix*. KCT-02 is an extended decoction after adding* Rubi Fructus* and* Salviae Miltiorrhizae Radix* to KCT-01 (Table 1). Although hepatoprotective effect of IJCGT and clinical case report have been demonstrated previously, scientific evidence to support the effect of KCT-01 or KCT-02 and their fundamental mechanism of action are currently unclear. Therefore, the objective of the present study was to compare anti-inflammatory effects of KCT-01, KCT-02, and IJCGT. After that, the most potent medicine among the three was selected to unravel the detailed molecular mechanism involved in its anti-inflammatory effect* in vitro* using LPS-induced RAW 264.7 macrophages and* in vivo* using croton oil-induced ear edema rat model.

## 2. Materials and Methods

### 2.1. Materials and Reagents

The herbal plants were purchased from Kyung Hee Herb Pharm (Wonju, Republic of Korea), a licensed company with Good Manufacturing Product (GMP) facilities. Briefly, 149.66 g of KCT-01, 198.34 g of KCT-02, and 307.13 g of IJCGT were extracted twice in 1500 ml, 2000 ml, and 3000 ml of 30% EtOH for 3 h at 80–85°C. After each extracted solution was processed through a filtration process, we could get 29.45 g of KCT-01 (yield = 19.63%), 44.26 g of KCT-02 (yield = 22.13%), and 56.36 g of IJCGT (yield = 18.78%) in forms of freeze-dried powders of each formula. HPLC-grade formic acid was purchased from Wako (Osaka, Japan) and HPLC-grade acetonitrile was obtained from Fisher Scientific Korea (Seoul, South Korea). Reference standards of chlorogenic acid, isochlorogenic acid A, isochlorogenic acid B, and ziyuglycoside I were purchased from Chemfaces (Wuhan, Hubei, China). Ellagic acid was purchased from Chromadex (Irvine, CA, USA) and hyperoside, jaceosidin, eupatilin, and glycyrrhizic acid were purchased from Chengdu Biopurify Phytochemicals Ltd. (Chengdu, Sichuan, China). Scoparone and luteolin were purchased from Sigma-Aldrich (St. Louis, MO, USA) and curcumin was purchased from HWI Analytik GmbH (Rheinzabern, Germany). Dulbecco's modified eagle's medium (DMEM), fetal bovine serum (FBS), and penicillin-streptomycin (PS) were obtained from Life Technologies Inc. (Grand Island, NY, USA). COX-2 antibody was purchased from Cayman (MI, USA), and phospho-STAT1 and phosphor-STAT3 antibodies were purchased from Cell Signaling Technology Inc. (Beverly, MA). iNOS, phospho-JAK1/2, JAK1/2, STAT1/3, and *β*-actin antibodies and peroxidase-conjugated secondary antibody were purchased from Santa Cruz Biotechnology, Inc. (CA, USA). The enzyme immunoassay [4] kits for PGE_2_, TNF-*α*, and IL-6 were obtained from R&D Systems (MN, USA). Random oligonucleotide primers and M-MLV reverse transcriptase were purchased from Promega (WI, USA). SYBR green ex Taq was purchased from TaKaRa (Shiga, Japan). COX-2, iNOS, TNF-*α*, IL-6, IFN-*β*, and *β*-actin oligonucleotide primers were obtained from Bioneer (Seoul, Korea). 3-(4,5-Dimethylthiazol-2-yl)-2,5-diphenyl tetrazolium bromide (MTT), NS-398, LPS (*Escherichia coli*, serotype 0111:B4), and all other chemicals were purchased from Sigma Chemical Co. (MO, USA).

### 2.2. UPLC-PDA-ESI-MS Analysis

A Waters Acquity™ H-class ultraperformance liquid chromatography (UPLC) system (Waters Corp., Milford, MA, USA) with photodiode array (PDA) detector and JMS-T100TD (AccuTOF-TLC) (JEOL Ltd., Tokyo, Japan) spectrometer equipped with electrospray ionization (ESI) source were used for chromatographic and spectrometric (MS) analysis. The chromatographic separation was carried out on an ACQUITY UPLC BEH C18 Column (130 Å, 1.7 *μ*m, 2.1 mm × 50 mm, Waters Corp., Milford, MA, USA) equipped with an ACQUITY UPLC BEH C18 VanGuard Precolumn (130 Å, 1.7 *μ*m, 2.1 mm × 5 mm). The mobile phase consisted of acetonitrile (solvent A) and 0.1% formic acid water (solvent B). The gradient condition of the mobile phase was 0–3 min, 5%; 3–5 min, 5% to 10%; 5–15 min, 10% to 15%; 15–20 min, 15% to 50%; 20–30 min, 50% to 100%; and 30–35 min, 100% as percent of solvent A. The flow rate was 0.6 mL/min and the column oven temperature was maintained at 40°C and the detection wavelength was 330 nm. The injection volume was 2.0 *μ*L. The conditions of MS analysis in the positive ion mode were as follows: scan range, *m*/*z* 50–1000; desolvating chamber temperature, 250°C; orifice 1 temperature, 80°C; orifice 1 voltage, 80 V; orifice 2 voltage, 10 V; ring lens voltage, 5 V; peak voltage, 1500 V; detector voltage, 2200 V; and nitrogen gas flow rate, 1.0 L/min (nebulizing gas) and 3.0 L/min (desolvating gas).

For the UPLC-MS analysis, ten milligrams of KCT-01 or IJCGT extract was dissolved in one milliliter of 30% ethanol or 70% ethanol, respectively. Reference standard compounds (1.0 mg/mL) were dissolved in methanol and then mixed into a cocktail solution which was used as reference standards solution. The sample solutions were filtered through a 0.2 *μ*m polyvinylidene fluoride syringe filter (Whatman, Maidstone, UK) and reference standards solutions were filtered through a 0.2 *μ*m polytetrafluoroethylene syringe filter (Whatman, Maidstone, UK) before injection into UPLC system.

### 2.3. Cell Culture and Sample Treatment

The RAW 264.7 murine macrophage cell line was purchased from the Korea Cell Line Bank (Seoul, Korea) and cultured in DMEM containing 10% fetal bovine serum, penicillin-streptomycin (100 units/mL) at 37°C with 5% CO_2_.

Murine bone marrow derived macrophages (BMDMs) were isolated from femur of C57BL/6 mice. Cells in bone marrow were washed several times with cold phosphate buffered saline (PBS). The isolated cells were filtered through sieve mesh, centrifuged, and resuspended in DMEM (supplemented with 10% FBS, 100 units/mL penicillin-streptomycin). The cells were incubated at 2 × 10^6^ in Petri dish with 15% L929-conditioned medium in DMEM for 7 days to differentiate into macrophages. The culture medium was added at the 3rd day and replaced at the 6th day of incubation. After being differentiated, the cells were seeded in 24-well culture plates. In all experiments, cells were incubated with samples at various concentrations that was always added 1 h prior to LPS (1 *μ*g/mL) treatment for the indicated time.

### 2.4. Animals

All experiments in the present study were conducted under university guideline of ethical committee for Animal Care and Use of the Kyung Hee University according to an animal protocol (KHUAPS(SE)-16-013). Male C57BL/6 mice (6–8 weeks old) or male Sprague-Dawley (SD) rats (6 weeks old) were purchased from the Orient Bio Inc. (Seongnam-si, Korea) and maintained under constant conditions (temperature: 20 ± 2°C, humidity: 40–60%, and light/dark cycle: 12 h).

### 2.5. Cell Viability Assay

Cell viability was evaluated by MTT assay. RAW 264.7 macrophage cells or BMDMs were plated at a density of 2 × 10^5^ cells per well in 24-well plates and then treated with samples (KCT-01, KCT-02, or IJCGT) at various concentrations 1 h prior to LPS (1 *μ*g/mL) treatment. After 24 h of LPS stimulation, 20 *μ*l MTT solution (5 mg/mL) was added to each well, and the cells were further incubated for an additional 4 h. The supernatant was removed and the formazan was resolved with 1 mL/well of DMSO. The optical density was measured at 540 nm by microplate reader.

### 2.6. Nitrite Determination

RAW 264.7 macrophage cells or BMDMs were plated at 2 × 10^5^ cells per well in 24-well plates and then incubated with or without LPS (1 *μ*g/mL) in the absence or presence of various concentrations of samples (KCT-01, KCT-02, or IJCGT) at various concentrations for 24 h. Nitrite levels in culture media were determined using the Griess reaction assay and presumed to reflect NO levels. The optical density was measured at 540 nm by microplate reader.

### 2.7. PGE_2_, TNF-*α*, and IL-6 Assay

RAW 264.7 macrophage cells or BMDMs were plated at 2 × 10^5^ cells per well in 24-well plates and then incubated with or without LPS (1 *μ*g/mL) in the absence or presence of various concentrations of samples. Dilutions of the cell culture medium were assayed for PGE_2_, TNF-*α*, IL-6, and IL-1*β*. PGE_2_ levels in cell culture medium were determined using a colorimetric competitive enzyme-linked immunosorbent assay (ELISA) kit (Enzo Life Science, NY, USA) according to manufacturer's instructions. TNF-*α* and IL-6 levels in cell culture medium were quantified using mouse DuoSet kit (R&D Systems, MN, USA) according to manufacturer's instructions.

### 2.8. Protein Extraction and Western Blot Analysis

RAW 264.7 macrophage cells were seeded in 60 mm^2^ dish and incubated for 24 h and then added to KCT-01 1 h prior to LPS (1 *μ*g/mL) treatment. The cells were collected by centrifugation and washed three times with PBS. Washed cell pellets were resuspended in protein extraction solution PROPREP (Intron Biotechnology, Seoul, Korea) and then incubated for 30 min at 4°C. Cell debris was removed by microcentrifugation and supernatants were quickly frozen. The protein concentration was determined using the Bio-Rad protein assay reagent (Bio-Rad Laboratories Inc., CA, USA) according to manufacturer's instruction. Proteins were electroblotted onto a PVDF membrane following separation on an 8% or 10% SDS-polyacrylamide gel electrophoresis. The immunoblot was incubated for 1 h with blocking solution (5% skim milk) at room temperature and then incubated overnight with a 1 : 1000 dilution of primary antibody at 4°C. Blots were washed three times with Tween 20/Tris-buffered saline (T/TBS) and then incubated with a 1 : 2000 dilution of horseradish peroxidase-conjugated secondary antibody (Santa Cruz Biotechnology Inc. CA, USA) for 2 h at room temperature. Blots were again washed three times with T/TBS and then developed by enhanced chemiluminescence (Amersham Life Science, IL, USA).

### 2.9. Total RNA Extraction and Quantitative Real-Time RT-PCR (qRT-PCR)

Total cellular RNA was isolated by Easy Blue kits (Intron Biotechnology, Seoul, Korea). 1 *μ*g of RNA was reverse-transcribed (RT) using MuLV reverse transcriptase, 1 mM deoxyribonucleotide triphosphate (dNTP), and 0.5 *μ*g/*μ*l oligo (dT_12–18_). Real-time PCR was performed using Thermal Cycler Dice Real-Time PCR System (Takara, Shiga, Japan). The primers used for SYBR green real-time reverse transcription-PCR were as follows: for* iNOS*, sense primer, 5′-CAT GCT ACT GGA GGT GGG TG-3′, antisense primer, 5′-CAT TGA TCT CCG TGA CAG CCC-3′; for* COX-2*, sense primer, 5′-TGC TGT ACA AGC AGT GGC AA-3′, antisense primer, 5′-GCA GCC ATT TCC TTC TCT CC-3′; for* TNF-α*, sense primer, 5′-AGC ACA GAA AGC ATG ATC CG-3′, antisense primer, 5′-CTG ATG AGA GGG AGG CCA TT-3′; for* IL-6*, sense primer, 5′-GAG GAT ACC ACT CCC AAC AGA CC-3′, antisense primer, 5′-AAG TGC ATC ATC GTT GTT CAT ACA-3′, for *β-actin*, sense primer, 5′-ATC ACT ATT GGC AAC GAG CG-3′, antisense primer, 5′-TCA GCA ATG CCT GGG TAC AT-3′. The results were expressed as the ratio of optimal density to *β*-actin.

### 2.10. Plasmid, Transient Transfection, and Luciferase Assay

RAW 264.7 macrophages were cotransfected with pNF-*κ*B-luc or pAP-1-luc (Clontech, Shiga, Japan) plasmid plus the phRL-TK plasmid (Promega, WI, USA) using Lipofectamine LTX™ (Invitrogen, CA, USA) as instructed by the manufacturers. After 24 h of transfections, cells were pretreated with KCT-01 for 1 h prior to LPS (1 *μ*g/mL) stimulation. After 18 h, each well was washed with cold-PBS and cells were lysed and the luciferase activity was determined using the Promega luciferase assay system (WI, USA).

### 2.11. Ear Edema Animal Model

Sprague-Dawley (SD) male rats weighing 180–200 g were divided into three groups (*n* = 6); croton-oil-alone group, KCT-01-50 mg/kg-treated group, and KCT-01-100 mg/kg-treated group. SD male rats were pretreated with KCT-01 (50 or 100 mg/kg, p.o.) and after 1 h, ear edema was induced on inner surface of the right ear by topical application of croton oil (5% solution in 100 *μ*L of acetone). The left ear was used as a control and received the same amount of the vehicle (acetone). Two hours after croton oil application, rats were sacrificed using CO_2_, and both ear tissues were collected using 6 mm punching. Ear punch biopsies were immediately measured for thickness to assess ear edema. Ear biopsies were fixed in 4% paraformaldehyde overnight and embedded in paraffin. Ear biopsy sections were stained with hematoxylin and eosin (H&E) at the Seoul Medical Science Institute (SCL Co. Ltd., Seoul, Korea). The other ear biopsies were immediately frozen (−70°C) for the determination of myeloperoxidase (MPO) activity, a marker of neutrophil influx into the tissue, and intracellular protein expression including iNOS, COX-2, and STATs. The tissue was thawed and homogenized. The homogenate was then centrifuged at 1500 ×g for 15 min, and the resulting supernatant was assayed for MPO assay and Western blotting.

### 2.12. Statistical Analysis

Data are presented as mean ± SD. Comparison between groups was made with SigmaPlot followed by Student's *t*-test. *p* values of 0.05 or less were considered statistically significant.

## 3. Results

### 3.1. Phytochemical Identification of KCT-01 and IJCGT by UPLC-PDA-ESI-MS

**Figure 1:**
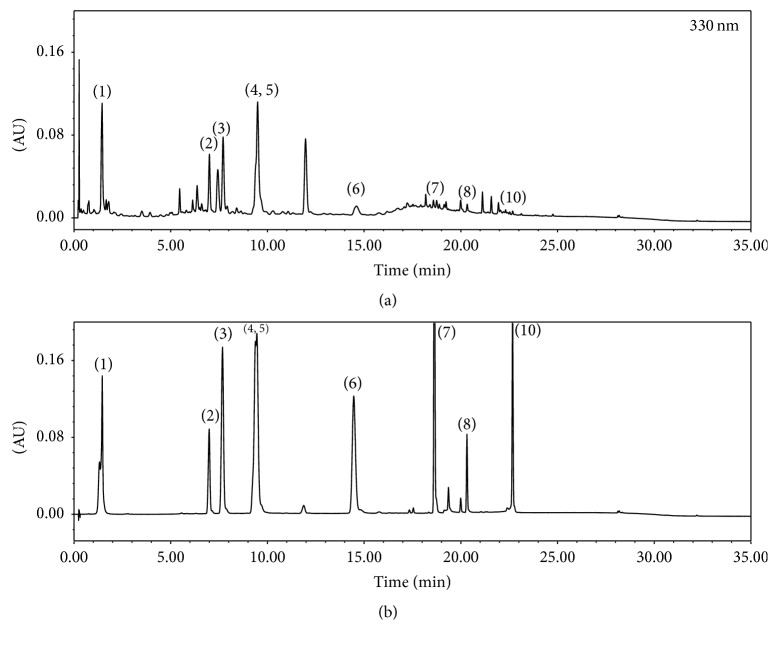
UPLC chromatograms of KCT-01 extract (a) and reference standards (b). (1) Chlorogenic acid; (2) hyperoside; (3) scoparone; (4) isochlorogenic acid A; (5) isochlorogenic acid B; (6) luteolin; (7) jaceosidin; (8) eupatilin; (9) ziyuglycoside I; and (10) curcumin.

**Figure 2:**
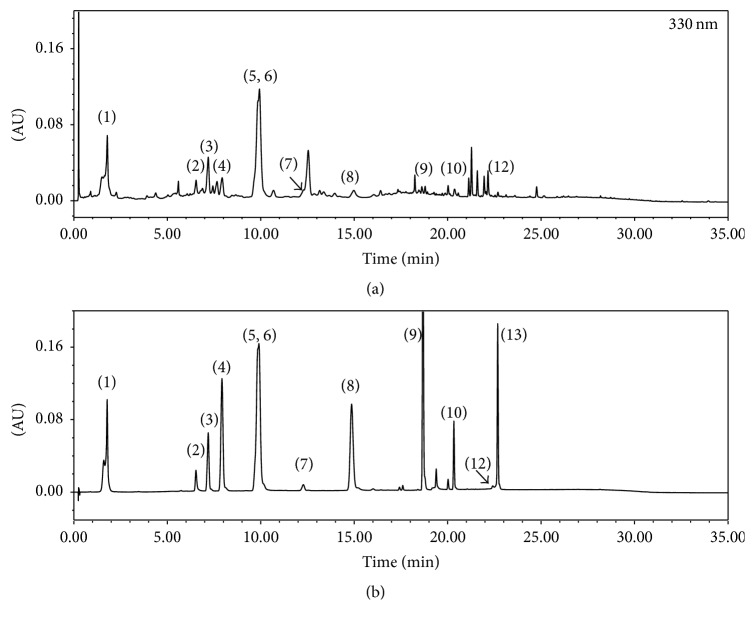
UPLC chromatograms of IJCGT extract (a) and reference standards (b). (1) Chlorogenic acid; (2) ellagic acid; (3) hyperoside; (4) scoparone; (5) isochlorogenic acid A; (6) isochlorogenic acid B; (7) hesperidin; (8) luteolin; (9) jaceosidin; (10) eupatilin; (11) ziyuglycoside I; (12) glycyrrhizic acid; (13) curcumin.

To identify phytochemicals of KCT-01 and IJCGT, chromatographic fingerprint analysis was carried out of KCT-01 or IJCGT by UPLC-PDA-MS and MS/MS. Chromatogram of KCT-01 detected at 330 nm is shown in Figure 1(a). Ten peaks (chlorogenic acid, hyperoside, scoparone, isochlorogenic acid A, isochlorogenic acid B, luteolin, jaceosidin, eupatilin, ziyuglycoside I, and curcumin) were confirmed by direct comparison with their corresponding reference standards (Figure 1, Supplementary Figure  1). Eleven peaks were identified in chromatogram of IJCGT shown in Figure 2(a) and Supplementary Figure  2(A). Qualitative identification using standard solutions confirmed the presence of chlorogenic acid, hyperoside, scoparone, isochlorogenic acid A, isochlorogenic acid B, luteolin, jaceosidin, eupatilin, ellagic acid, ziyuglycoside I, and glycyrrhizic acid (Figures 1(b) and 2(b), Supplementary Figures  1(B) and 2(B)). Chlorogenic acid, hyperoside, scoparone, isochlorogenic acid A, isochlorogenic acid B, luteolin, jaceosidin, and eupatilin have been previously identified in* Artemisia Capillaris *[22]. Ziyuglycoside I in* Sanguisorba officinalis *[23], curcumin in* Curcuma longa *[24], ellagic acid in* Rubus coreanus *[25], hesperidin in* Citrus unshiu *[26], ziyuglycoside I in* Sanguisorba officinalis *[23], and glycyrrhizic acid in* Glycyrrhiza uralensis *[27] have been previously identified. Detailed UPLC-MS data of these peaks are listed in Tables 2 and 3. Based on UPLC-PDA-MS analysis, representative phytochemicals from each herbal formulation were successfully identified.

### 3.2. Effect of KCT-01, KCT-02, or IJCGT on NO and PGE_2_ Production, and Cell Viability in LPS-Induced RAW 264.7 Macrophages

**Figure 3:**
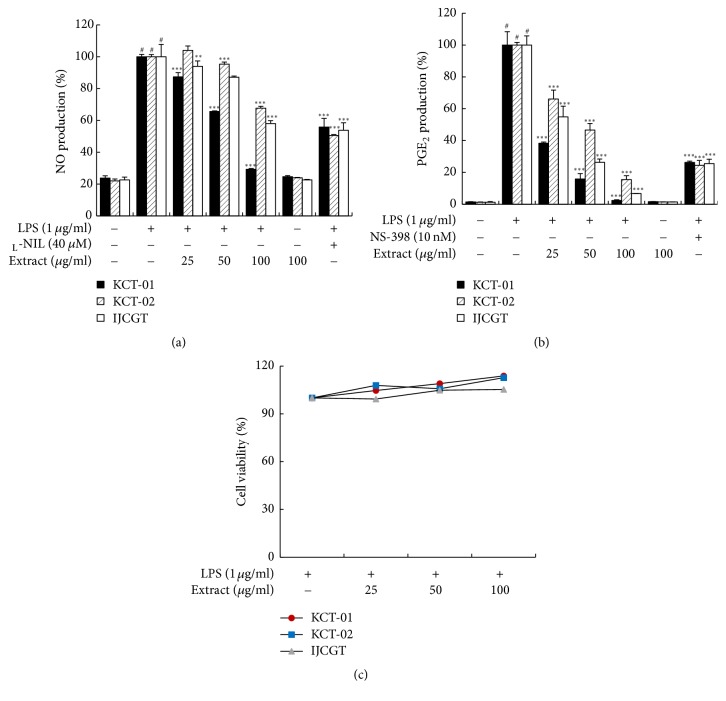
KCT-01, KCT-02, or IJCGT inhibited production of NO and PGE_2_ in LPS-induced RAW264.7 macrophages at noncytotoxic concentrations. Cells were treated with KCT-01, KCT-02, or IJCGT at various concentrations (6.25–100 *μ*g/mL) plus LPS (1 *μ*g/mL) or LPS alone for 24 h. (a) Cell viability was measured by MTT assay and NO production was measured using Griess reaction assay. (b) PGE_2_ production was measured using an EIA kit. Values are expressed as means ± SD of three independent experiments. ^#^*p* < 0.05 versus control, ^*∗∗*^*p* < 0.01 and ^*∗∗∗*^*p* < 0.001 versus LPS-treated cells.

To evaluate inhibitory properties of KCT-01, KCT-02, or IJCGT on production of NO and PGE_2_, RAW 264.7 macrophages were pretreated with various concentrations (25, 50, or 100 *μ*g/mL) of KCT-01, KCT-02, or IJCGT for 24 h in presence of LPS (1 *μ*g/mL). As shown in Figure 3(a) and Table 4, KCT-01, KCT-02, and IJCGT each suppressed NO production in a concentration-dependent manner. At concentration of 100 *μ*g/mL, KCT-01, KCT-02, and IJCGT suppressed NO production by 70.71%, 32.31%, and 42.02%, respectively (IC_50_ of KCT-01, KCT-02, and IJCGT: 64.93 *μ*g/mL, >100 *μ*g/mL, and >100 *μ*g/mL, resp.). At concentration of 100 *μ*g/mL, KCT-01, KCT-02, and IJCGT also suppressed PGE_2_ production by 97.67%, 84.47%, and 93.22%, respectively (IC_50_: 18.18 *μ*g/mL, 51.75 *μ*g/mL, and 27.70 *μ*g/mL, resp.) (Figure 3(b)). _L_-NIL (40 *μ*M) and NS398 (10 nM) were used as NO and PGE_2_ inhibitors, respectively. As shown in Table 4, KCT-01 more effectively blocked production of NO and PGE_2_ than KCT-02 or IJCGT. The potentially cytotoxic effect of KCT-01, KCT-02, or IJCGT on RAW 264.7 macrophages was determined by MTT assay. Results showed that viability of cells was not significantly affected by KCT-01, KCT-02, or IJCGT at concentrations up to 100 *μ*g/mL (Figure 3(c)), indicating that their suppressive effects on NO and PGE_2_ production were not attributable to their nonspecific cytotoxicity.

### 3.3. Effect of KCT-01, KCT-02, or IJCGT on Production of TNF-*α* and IL-6 in LPS-Induced RAW 264.7 Macrophages

**Figure 4:**
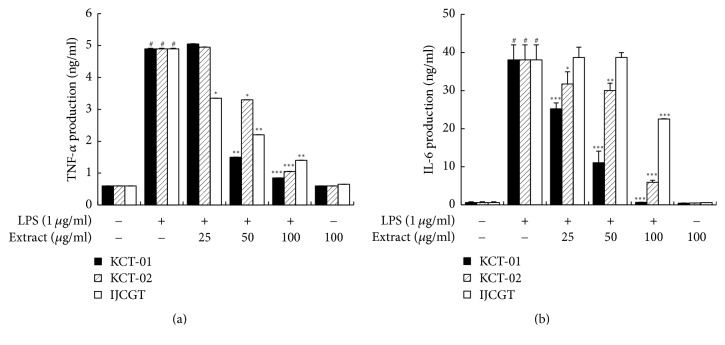
Effects of KCT-01, KCT-02, or IJCGT on proinflammatory cytokines production in LPS-induced RAW264.7 macrophages. Cells were treated with KCT-01, KCT-02, or IJCGT at various concentrations (12.5–100 *μ*g/mL) plus LPS (1 *μ*g/mL) or LPS alone for 24 h. (a) Productions of TNF-*α* and IL-6 were measured using EIA kits. Values are expressed as means ± SD of three independent experiments. ^#^*p* < 0.05 versus control; ^*∗*^*p* < 0.05, ^*∗∗*^*p* < 0.01, and ^*∗∗∗*^*p* < 0.001 versus LPS-treated cells.

TNF-*α* and IL-6 are important inflammatory cytokines secreted by macrophages [28]. Therefore, we examined effects of KCT-01, KCT-02, or IJCGT on production of proinflammatory cytokines (TNF-*α* and IL-6) in LPS-induced RAW 264.7 macrophages. Cells were pretreated with KCT-01, KCT-02, or IJCGT at various concentrations (25, 50, or 100 *μ*g/mL) for 1 h followed by stimulation with LPS for 24 h. KCT-01, KCT-02, or IJCGT each concentration dependently and significantly suppressed the production of TNF-*α* and IL-6 (Figure 4). Of these three, KCT-01 showed the most potent inhibition for TNF-*α* (IC_50_: 22.9 *μ*g/mL) and IL-6 (IC_50_: 35.5 *μ*g/mL) production. Collectively, our results indicate that KCT-01 is a more potent herbal medicine that can suppress proinflammatory mediators compared to KCT-02 or IJCGT (Table 4). Therefore, we selected KCT-01 as the most effective anti-inflammatory agent and conducted further experiments to determine the underlying molecular mechanism involved in its anti-inflammatory effect.

### 3.4. KCT-01 Inhibits Production of NO, PGE_2_, TNF-*α*, and IL-6 in LPS-Induced BMDMs

**Figure 5:**
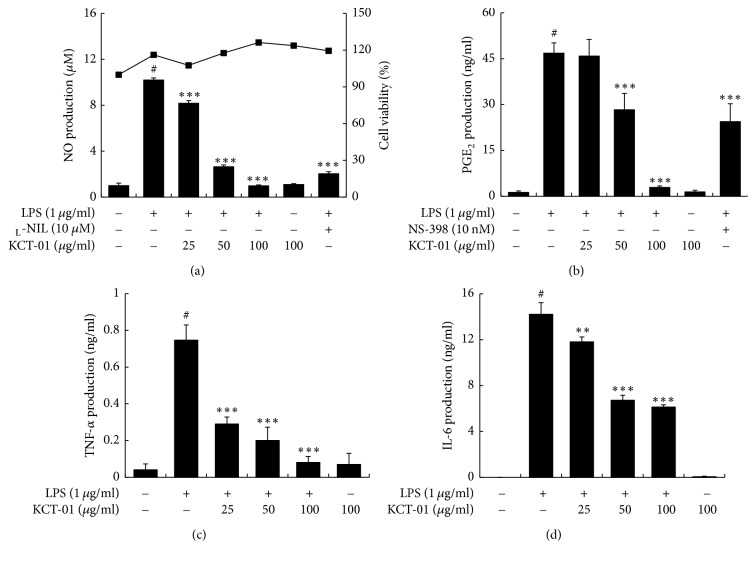
KCT-01 inhibited NO, PGE_2_, TNF-*α*, and IL-6 productions in LPS-induced mouse bone marrow derived macrophages. Cells were treated with KCT-01, KCT-02, or IJCGT at various concentrations (25–100 *μ*g/mL) plus LPS (1 *μ*g/mL) or LPS alone for 24 h. (a) Cell viability was measured by MTT assay and NO production was measured using Griess reaction. ((b), (c), and (d)) PGE_2_, TNF-*α*, and IL-6 productions were measured by EIA kit. Values are expressed as means ± SD of three independent experiments. ^#^*p* < 0.05 versus control; ^*∗∗*^*p* < 0.01 and ^*∗∗∗*^*p* < 0.001 versus LPS-treated cells.

To confirm our findings in primary macrophage cells, we examined the inhibitory effect of KCT-01 on production of proinflammatory mediators and cytokines in LPS-induced BMDMs. We found that KCT-01 significantly and concentration dependently inhibited production of NO, PGE_2_, TNF-*α*, and IL-6 in LPS-stimulated BMDMs (Figure 5), indicating that the anti-inflammatory effect of KCT-01 might not be cell specific responses.

### 3.5. KCT-01 Inhibits Expression Levels of iNOS, COX-2, TNF-*α*, and IL-6 in LPS-Induced RAW 264.7 Macrophages

**Figure 6:**
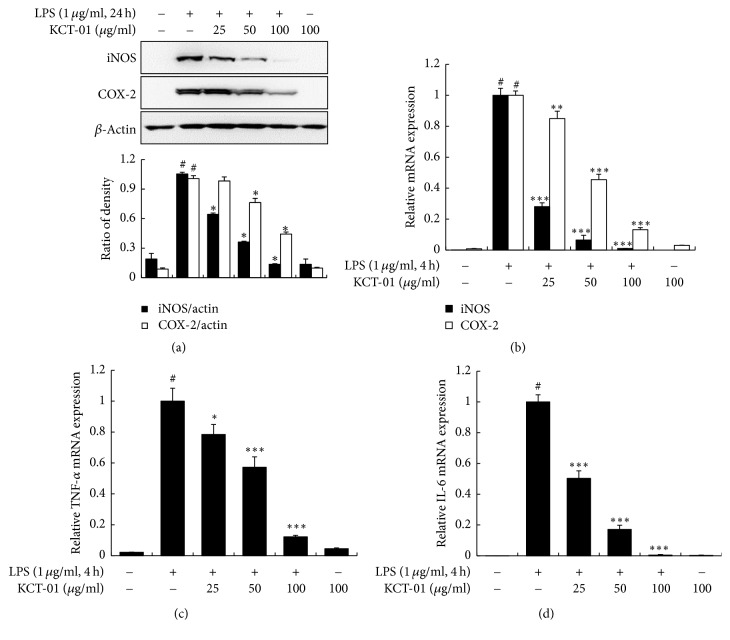
KCT-01 inhibits the expression of inflammatory genes in LPS-induced RAW264.7 macrophages. Cells were treated with KCT-01 plus LPS (1 *μ*g/mL) or LPS alone for indicated time. (a) Total cellular proteins were prepared and subjected to Western blotting to determine protein expression levels of iNOS and COX-2. Levels of iNOS and COX-2 were normalized against *β*-actin expression. ((b), (c), and (d)) Total cellular RNA were prepared and subjected to qRT-PCR to determine mRNA expression levels of iNOS, COX-2, TNF-*α*, and IL-6. Levels of iNOS, COX-2, TNF-*α*, and IL-6 were normalized against *β*-actin expression. Values are expressed as means ± SD of three independent experiments. ^#^*p* < 0.05 versus control; ^*∗*^*p* < 0.05, ^*∗∗*^*p* < 0.01, and ^*∗∗∗*^*p* < 0.001 versus LPS-treated cells.

Next, we evaluated whether the inhibitory effect of KCT-01 on the production of NO and PGE_2_ was related to reduced expression of iNOS and COX-2 by Western blotting and qRT-PCR. As shown in Figure 6(a), iNOS and COX-2 protein levels were markedly increased by LPS. However, KCT-01 significantly suppressed these upregulations in a concentration-dependent manner. KCT-01 also markedly inhibited LPS-induced iNOS and COX-2 mRNA expression levels (Figure 6(b)). These findings demonstrate that KCT-01 can downregulate the expression of LPS-induced iNOS and COX-2 and lead to decreased production of NO and PGE_2_. KCT-01 also downregulated TNF-*α* and IL-6 mRNA expression levels in a concentration-dependent manner (Figures 6(c) and 6(d)). Taken together, these results suggest that KCT-01 possesses anti-inflammatory activity by inhibiting expression levels of various LPS-induced proinflammatory mediators.

### 3.6. Effects of KCT-01 on NF-*κ*B and AP-1 Activation in LPS-Induced RAW 264.7 Macrophages

**Figure 7:**
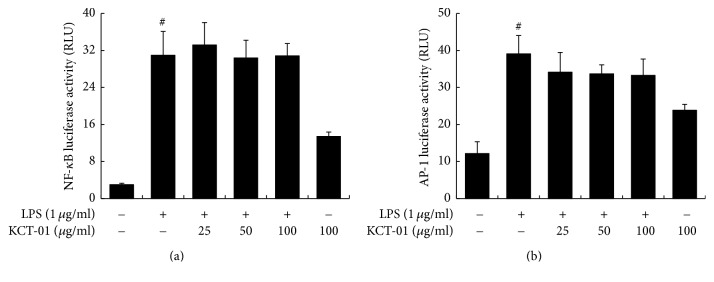
KCT-01 has no inhibitory effect on activation of NF-*κ*B or AP-1 in LPS-induced RAW264.7 macrophages. Cells were transfected with p-NF-*κ*B-luc reporter or pAP-1-luc reporter. phRL-TK vector was used as an internal control. Cells were then treated with KCT-01 plus LPS or LPS alone. After 18 h of treatment, luciferase activity levels were determined using luciferase assay. Values are expressed as the means ± SD of three independent experiments. ^#^*p* < 0.05 versus control.

NF-*κ*B and AP-1 are key transcriptional factors regulating inflammatory responses mediated by LPS or proinflammatory cytokines [29, 30]. Therefore, we explored the effect of KCT-01 on LPS-induced NF-*κ*B or AP-1 activity in RAW 264.7 macrophages by luciferase reporter gene assay using pNF-*κ*B-luc or pAP-1-luc vector. Our results revealed that KCT-01 did not have any effect on NF-*κ*B or AP-1-dependent transcriptional activity (Figure 7). When transcription factors are phosphorylated, these may translocate to the nucleus where they can bind to their consensus DNA binding sites to regulate transcription of target genes [31]. Accordingly, we performed Western blotting to determine the effect of KCT-01 on phosphorylation of p65 (a subunit of NF-*κ*B) or c-fos and c-jun (subunits of AP-1). Our results showed that KCT-01 did not have any inhibitory effect on LPS-induced phosphorylation of p65, c-fos, and c-jun (data not shown).

### 3.7. Effect of KCT-01 on Activation of JAK/STAT Signaling Pathway in LPS-Induced RAW 264.7 Macrophages

**Figure 8:**
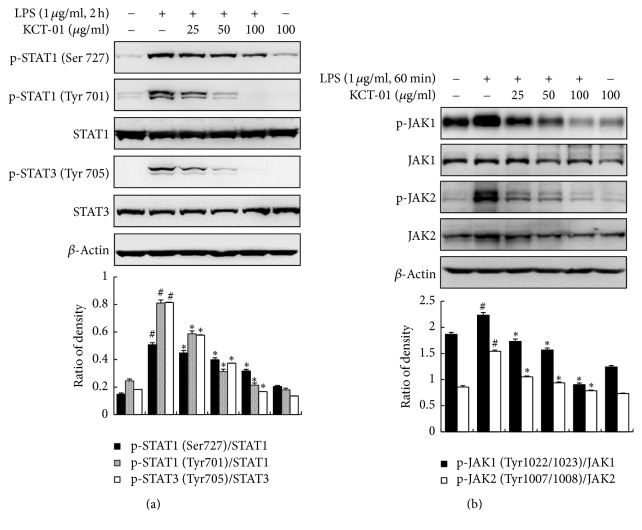
KCT-01 suppresses activation of JAK/STAT signaling cascades in LPS-induced RAW264.7 macrophages. Cells were treated with KCT-01 plus LPS (1 *μ*g/mL) or LPS alone for indicated time. (a) Total cellular proteins were prepared and subjected to Western blotting to determine phosphorylation levels of STAT1 (Ser727 or Tyr 701), STAT3 (Tyr705), and JAK1/2. Levels of STATs and JAKs were normalized against *β*-actin expression. Values are expressed as means ± SD of three independent experiments. ^#^*p* < 0.05 versus control; ^*∗*^*p* < 0.05 versus LPS-treated cells.

JAK/STAT signaling pathway is involved in immunity and has also affected inflammatory signaling cascades triggered by LPS, IFN-*γ*, and other cytokines [7, 32]. Since KCT-01 had no effect on NF-*κ*B and AP-1 activation, we investigated whether the inhibitory effect of KCT-01 on the expression of proinflammatory mediators was mediated by JAK/STAT signaling pathway. Binding of LPS ligands to their receptors induces phosphorylation of receptor-associated JAKs followed by phosphorylation of STATs [9]. As shown in Figure 8(a), KCT-01 concentration dependently downregulated the phosphorylation of STAT1 (Ser727 and Tyr701) and STAT3 (Tyr705) at 2 h after LPS stimulation. KCT-01 also significantly and concentration dependently blocked the phosphorylation of JAK1 (Tyr1022/1023) and JAK2 (Tyr1007/1008) at 1 h after LPS challenge (Figure 8(b)). Therefore, KCT-01 potently inhibited JAK1 and JAK2, subsequently downregulating phosphorylation of STAT1 and STAT3.

### 3.8. Effect of KCT-01 on Croton Oil-Induced Ear Edema in Rats

**Figure 9:**
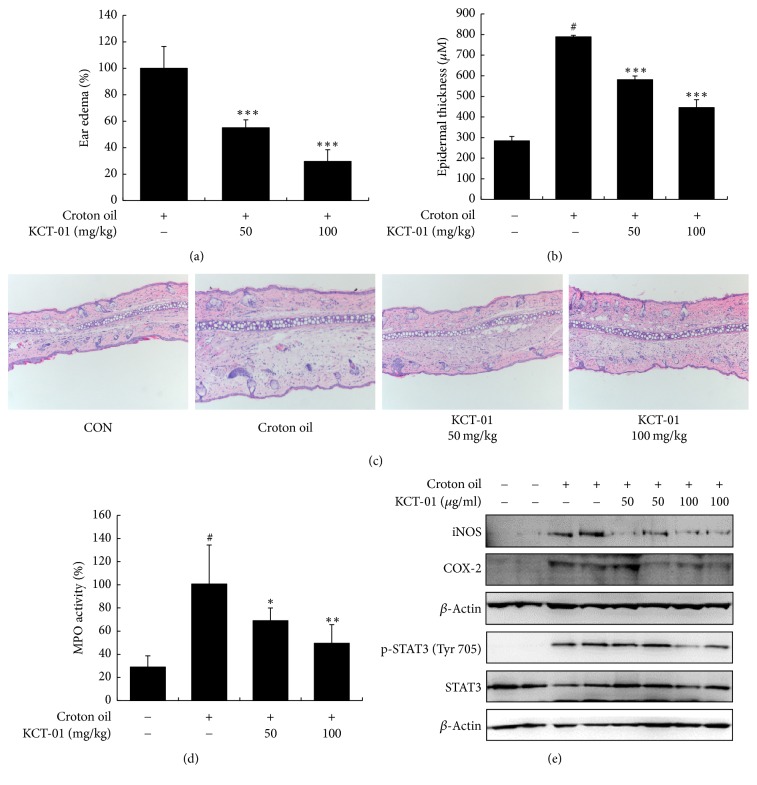
KCT-01 inhibits ear inflammation induced by croton oil in rats. (a) KCT-01 (50 or 100 mg/kg, p.o.) was administered 1 h prior to croton oil treatment and 6 mm ear punch was measured for thickness at 3 h after croton oil application to assess ear edema. ((b) and (c)) Epidermal thickness of ear tissues and histological analysis of croton oil-induced ear edema. Representative H&E sections of ear tissues were obtained from vehicle-treated control rat's left ear (control), croton oil treated rat's right ear, and KCT-01 (50 or 100 mg/kg, p.o.) plus croton oil treated rat's right ear. ((d) and (e)) Ear tissues were homogenized and lysates were used to determine MPO activity, protein expression, and phosphorylation of iNOS, COX-2, and STAT3. Values are expressed as means ± SD. ^#^*p* < 0.05 versus control; ^*∗*^*p* < 0.05, ^*∗∗*^*p* < 0.01, and ^*∗∗∗*^*p* < 0.001 versus croton oil treated group.

To determine the anti-inflammatory effect of KCT-01* in vivo*, we used a rat model of acute inflammatory ear edema induced by croton oil. As expected, ear thickness increased by croton oil application was reduced by pretreatment with KCT-01. At 50 and 100 mg/kg, KCT-01 decreased ear thickness by 45.00 ± 6.08% and 70.45 ± 8.86%, respectively (Figure 9(a)). To examine histopathological changes during ear edema, cross sections of ear discs were stained with hematoxylin and eosin. Consistent with its inhibitory effect on ear thickness, KCT-01 also inhibited epidermal ear edema after pretreatment (Figures 9(b) and 9(c)). Next, neutrophil migration into croton oil-induced ear was indirectly determined using MPO activity assay. As expected, application of croton oil to ear increased MPO production in tissues. However, oral administration of KCT-01 suppressed croton oil-induced MPO production (Figure 9(d)). Consistent with our findings in macrophages, KCT-01 also inhibited iNOS and COX-2 expression levels and STAT3 phosphorylation (Figure 9(e)).

## 4. Discussion

Many studies have reported that traditional herbal resources can benefit the management of various diseases, including arthritis [33], atopic dermatitis [34], and hepatic fibrosis [35]. According to classic literature such as well-known herbal medical books “Sang han lon” and “Geum gwe yo lyag,” a variety of herbal medications have been used to treat many diseases in Asian countries. In particular, “Geum gwe yo lyag” recommended IJORS (injinoryung-san), a herbal decoction consisting of* Artemisia Capillaris Herba*,* Alismatis Rhizoma*,* Poria Sclerotium*,* Atractylodis Macrocephalae Rhizoma*,* Polyporus Sclerotium*, and* Cinnamomi Cortex* as a typical prescription to treat jaundice. Based on IJORS expelling jaundice, IJCGT is made by omitting* Cinnamomi Cortex* but adding* Sanguisorbae Radix*,* Rubi Fructus*,* Glycyrrhizae Radix*,* Raphani Semen*, and* Citrus Unshiu Immaturi Pericarpium*, and* Zingiberis Rhizoma Crudus *(Table 1). Currently, IJCGT is mainly applied to treat patients with various liver diseases, such as viral hepatitis, cirrhosis, and hepatocellular carcinoma in Kyung Hee University Korean Medicine Hospital (Seoul, Korea). Among 11 individual medicines,* Artemisia Capillaris Herba *and* Sanguisorbae Radix *are regarded as key components of IJCGT for its various pharmacological effects for hepatic diseases, including liver cirrhosis and hepatoma usually caused by chronic inflammation followed by pathological changes in liver function [17, 36–40]. In addition, increasing evidence has revealed that curcumin, an active ingredient of* Curcuma longa Radix, *possesses anti-inflammatory properties [41, 42]. Thus, we developed a compressed herbal prescription consisting of* Artemisia Capillaris Herba, Sanguisorbae Radix*, and* Curcuma longa Radix* to treat hepatic inflammation. It was named KCT-01. Additionally, KCT-02 was expanded from KCT-01 by adding* Rubi Fructus *and* Salviae Miltiorrhizae Radix* to strengthen its anti-inflammatory activities [20, 43, 44]. In the present study, we evaluated anti-inflammatory effects of KCT-01, KCT-02, and IJCGT and elucidated underlying molecular mechanisms of KCT-01 in LPS-induced RAW 264.7 macrophages. Based on their inhibitory potencies on LPS-induced NO, PGE_2_, TNF-*α*, and IL-6 production, we selected KCT-01 to further investigate its anti-inflammatory effects and underlying molecular mechanism* in vitro* (LPS-induced macrophages) and* in vivo* (croton oil-induced acute inflammation in ear edema of rat).

LPS, an inflammatory stimulator, can induce various proinflammatory mediators and cytokines. Our data revealed that proinflammatory mediators (NO, PGE_2_) and cytokines (TNF-*α*, IL-6) were induced by LPS stimulation in RAW 264.7 macrophages. However, KCT-01, KCT-02, and IJCGT attenuated the expression of NO, PGE_2_, TNF-*α*, and IL-6 without causing cytotoxicity. These results indicate that KCT-01, KCT-02, and IJCGT exhibit anti-inflammatory effects on LPS-induced RAW 264.7 macrophages. We selected KCT-01 as the most potent prescription/formulation among the three and elicited molecular mechanisms involved. The inhibitory effect of KCT-01 on the release of NO, PGE_2_, TNF-*α*, and IL-6 was also proved in murine bone marrow derived macrophages. NO and PGE_2_ are major proinflammatory mediators produced by enzymes iNOS and COX-2, respectively. These two enzymes are responsible for cell damage and tissue destruction in inflammation [45, 46]. Consistently, we observed that KCT-01 also inhibited mRNA expression of iNOS, COX-2, TNF-*α*, and IL-6, indicating that KCT-01 downregulated the expression of inflammatory genes at transcription level.

To further explore the intracellular mechanism underlying the anti-inflammatory effect of KCT-01, we focused on various transcription factors such as NF-*κ*B, AP-1, and STATs involved in the regulation of inflammatory genes. Mechanistically, LPS induces TLR4 to activate NF-*κ*B, an important transcription factor for iNOS and COX-2 expression in various cells including macrophages [47]. LPS can also activate AP-1 which is stimulated by mitogen-activated protein kinases (MAPKs), including ERK1/2, p38 MAPKs, and JNK, thus enhancing proinflammatory gene expression in macrophages [48–50]. However, KCT-01 did not influence NF-*κ*B or AP-1 activation. Besides NF-*κ*B and AP-1 signaling, some studies have reported that the JAK/STATs signal pathway is crucial for the expression of genes encoding inflammatory enzymes such as iNOS and COX-2 [5, 8, 9]. The JAK-STATs pathway is also critical for cytokine activated signaling in immune response [51–53]. Thus, it is reasonable to speculate that the inhibitory effect of KCT-01 on LPS-stimulated inflammatory response might be attributed to its suppression on JAK-STATs signaling pathway. Accordingly, we found that KCT-01 blocked phosphorylation of both JAK and STATs in a concentration-dependent manner which in turn inhibited translocation of STATs to the nucleus to bind to promoters of target genes for their transcription activation [6]. These results demonstrated that KCT-01 could restrain LPS-elevated release of proinflammatory mediators via blocking STAT1 and STAT3 activation.

To verify whether KCT-01 could ameliorate acute inflammatory symptoms* in vivo*, we used croton oil-induced ear edema rat model. As expected, we found that oral administration of KCT-01 significantly suppressed the swelling of ears and MPO activity in ear tissues, indicating that KCT-01 could inhibit acute inflammation via inhibiting infiltration of inflammatory cells. Consistent with our findings in macrophages, KCT-01 inhibited protein expression of inflammatory mediators (including iNOS and COX-2) and phosphorylation of STAT3 in ear tissues.

Herbal medicine has limitations in practical uses as a drug because it is difficult to standardize herbal medicine or find obvious active components of each formulation. Thus, most herbal medicines are underestimated although they are effective in treating various diseases. As part of our effort to standardize herbal medicine, we analyzed phytochemicals of KCT-01 and IJCGT using chromatographic fingerprint analysis. However, we need to investigate active components of KCT-01. If we can scientifically verify and prove therapeutic effects of this herbal medicine, the value of KCT-01 will be increased.

## 5. Conclusions

In conclusion, we found that KCT-01, KCT-02, and IJCGT suppressed inflammatory mediators, with KCT-01 being the most effective one among the three. We also proved that the inhibitory effect of KCT-01 on inflammatory proteins and genes was accompanied by suppression of phosphorylation of JAK1/2 and STAT1/3. Therefore, this study provides evidence that KCT-01 might exhibit anti-inflammatory effect via suppressing JAK/STATs activation in LPS-induced RAW 264.7 macrophages. Such mechanism of action also contributed to the pharmacological potential of KCT-01* in vivo *using croton oil-induced ear edema model. Our findings suggest that KCT-01 may have potential as a herbal medicine for treating a variety of inflammatory diseases.

## Figures and Tables

**Table 1:** Prescription of KCT-01, KCT-02, and IJCGT.

Formula	Scientific name
KCT-01	*Artemisia Capillaris Herba, Sanguisorbae Radix, *and *Curcuma longa Radix*
KCT-02	*Artemisia Capillaris Herba, Sanguisorbae Radix,Curcuma longa Radix, Rubi Fructus,* and *Salviae Miltiorrhizae Radix*
IJCGT	*Artemisia Capillaris Herba*, *Sanguisorbae Radix*, *Rubi Fructus*, *Atractylodis Rhizoma Alba*, *Poria Sclerotium*, *Polyporus Sclerotium, Alismatis Rhizoma*, *Glycyrrhizae Radix*, *Raphani Semen*, *Citrus Unshiu Immaturi Pericarpium*, and *Zingiberis Rhizoma Crudus*

**Table 2:** Retention times (Rt), precursor ions, molecule weights, and UV Maxima (*λ* max) of identified peaks of KCT-01.

Compound	Rt (min)	Precursor ion (*m*/*z*)	Molecule weight	*λ* max (nm)
(1) Chlorogenic acid	1.78	355.10 [M+H]^+^	354.10	218, 242, 325
		377.08 [M+Na]^+^		
(2) Hyperoside	7.19	465.10 [M+H]^+^	464.10	203, 255, 353
(3) Scoparone	7.92	207.06 [M+H]^+^	206.06	203, 229, 343
(4) Isochlorogenic acid A	9.90	517.13 [M+H]^+^	516.13	218, 244, 327
(5) Isochlorogenic acid B	9.90	517.14 [M+H]^+^	516.13	218, 244, 327
(6) Luteolin	14.86	287.06 [M+H]^+^	286.05	253, 349
(7) Jaceosidin	18.68	331.08 [M+H]^+^	330.07	214, 272, 345
(8) Eupatilin	20.33	345.10 [M+H]^+^	344.09	214, 273, 343
(9) Ziyuglycoside I	20.61	767.46 [M+H]^+^	766.45	
		784.48 [M+NH_4_]^+^		
(10) Curcumin	22.68	369.13 [M+H]^+^	368.12	197, 264, 428

**Table 3:** Retention time (Rt), precursor ion, molecule weight, and UV Maxima (*λ* max) of identified peaks of IJCGT.

Compound	Rt (min)	Precursor ion (*m*/*z*)	Molecule weight	*λ* max (nm)
(1) Chlorogenic acid	1.78	355.10003 [M+H]^+^	354.10	218, 242, 325
		377.08276 [M+Na]^+^		
(2) Ellagic acid	6.53	303.02 [M+H]^+^	302.01	196, 253, 366
(3) Hyperoside	7.19	465.11 [M+H]^+^	464.10	203, 255, 353
(4) Scoparone	7.92	207.06 [M+H]^+^	206.06	203, 229, 343
(5) Isochlorogenic acid A	9.90	517.14 [M+H]^+^	516.13	218, 244, 327
(6) Isochlorogenic acid B	9.90	517.13 [M+H]^+^	516.13	218, 244, 327
(7) Hesperidin	12.28	611.20 [M+H]^+^	610.19	199, 283, 329
(8) Luteolin	14.86	287.06 [M+H]^+^	286.05	253, 349
(9) Jaceosidin	18.68	331.08 [M+H]^+^	330.07	214, 272, 345
(10) Eupatilin	20.33	345.10 [M+H]^+^	344.09	214, 273, 343
(11) Ziyuglycoside I	20.61	767.46 [M+H]^+^	766.45	
		784.49 [M+NH_4_]^+^		
(12) Glycyrrhizic acid	21.49	823.41 [M+H]^+^	822.40	252

**Table 4:** Effect of KCT-01, KCT-02, or IJCGT on NO, PGE_2_, TNF-*α*, and IL-6 production in LPS-induced RAW264.7 macrophages.

	IC_50_^ ^^a^ (*μ*g/ml)				
	Cell viability	NO	PGE_2_	TNF-*α*	IL-6
KCT-01	>100	64.93 ± 0.77	18.18 ± 2.73	22.88 ± 0.96	35.47 ± 3.77
KCT-02	>100	>100	51.75 ± 3.02	31.78 ± 4.59	57.93 ± 3.31
IJCGT	>100	>100	27.70 ± 1.94	48.14 ± 6.71	>100

^a^Data are presented as means ± SD of three independent experiments.

## Supplementary Materials

Supplementary Materials Figure S1. UPLC chromatograms of KCT-01 (A) and reference standards (B) at 203 nm. 1, chlorogenic acid; 2, hyperoside; 3, scoparone; 4, isochlorogenic acid A; 5, isochlorogenic acid B; 6, luteolin; 7, jaceosidin; 8, eupatilin; 9, ziyuglycoside I; 10, curcumin. Figure S2. UPLC chromatograms of IJCGT extract (A) and reference standards (B) at 203 nm. 1, chlorogenic acid; 2, ellagic acid; 3, hyperoside; 4, scoparone; 5, isochlorogenic acid A; 6, isochlorogenic acid B; 7, hesperidin; 8, luteolin; 9, jaceosidin; 10, eupatilin; 11, ziyuglycoside I; 12, glycyrrhizic acid; 13, curcumin.
