# Supplementary figures and images for: Kyungheechunggan-Tang-01, a New Herbal Medication, Suppresses LPS-Induced Inflammatory Responses through JAK/STAT Signaling Pathway in RAW 264.7 Macrophages
Source: Evid Based Complement Alternat Med. 2017 Nov 29;2017:7383104. doi: 10.1155/2017/7383104 (PMC5733936; doi:10.1155/2017/7383104)

**Supplementary material**

**Figure S1.**

**
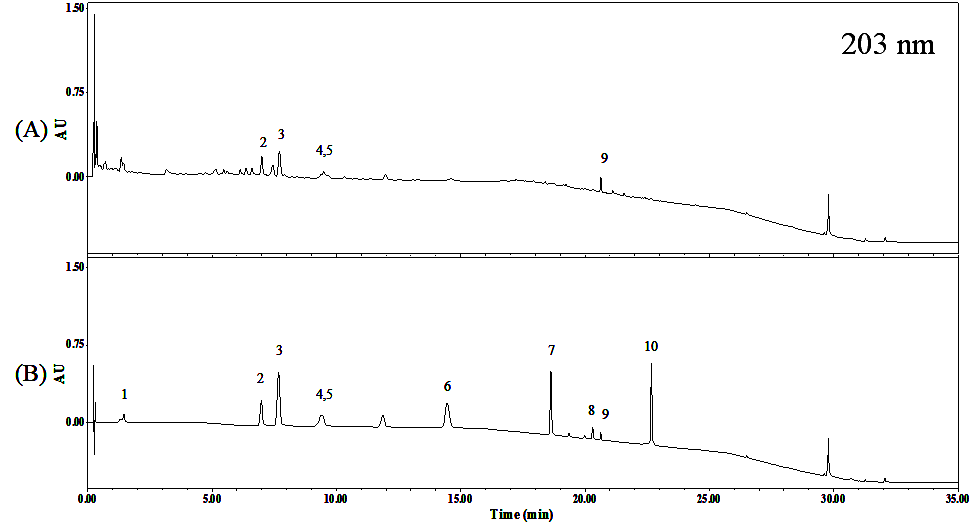
**

**Figure S2.**

**
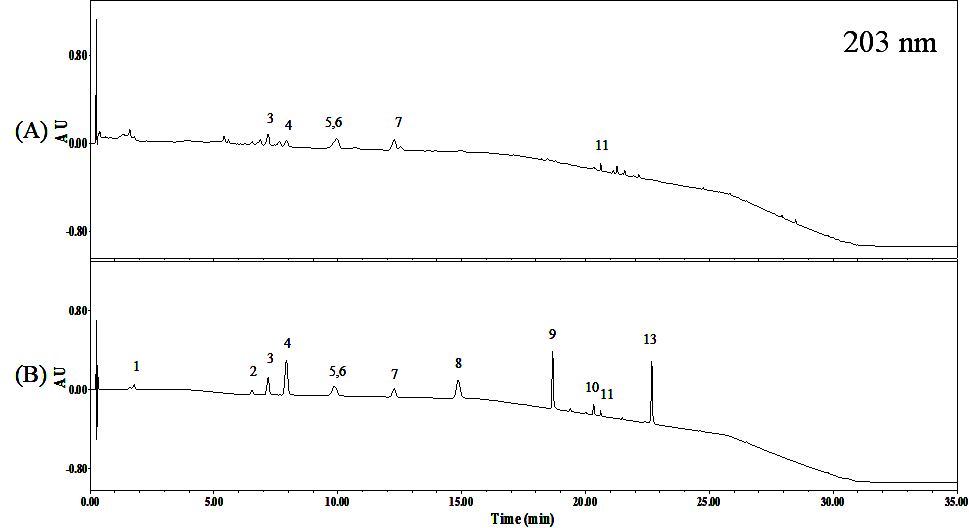
**

Supplement: Supplementary Materials — Figure S1. UPLC chromatograms of KCT-01 (A) and reference standards (B) at 203 nm. 1, chlorogenic acid; 2, hyperoside; 3, scoparone; 4, isochlorogenic acid A; 5, isochlorogenic acid B; 6, luteolin; 7, jaceosidin; 8, eupatilin; 9, ziyuglycoside I; 10, curcumin. Figure S2. UPLC chromatograms of IJCGT extract (A) and reference standards (B) at 203 nm. 1, chlorogenic acid; 2, ellagic acid; 3, hyperoside; 4, scoparone; 5, isochlorogenic acid A; 6, isochlorogenic acid B; 7, hesperidin; 8, luteolin; 9, jaceosidin; 10, eupatilin; 11, ziyuglycoside I; 12, glycyrrhizic acid; 13, curcumin. [file 7383104.f1.docx]
